# Colistin-stabilized antisolvent precipitation enables engineering of microcrystalline niclosamide for inhalable composite powders

**DOI:** 10.1016/j.ijpharm.2026.126791

**Published:** 2026-03-19

**Authors:** Mariana Romero-Gonzalez, Mari Park, Ziting Chen, Grace Xia, Bhanuz Dechayont, Ashlee D. Brunaugh

**Affiliations:** a Department of Pharmaceutical Sciences, University of Michigan, Ann Arbor, MI 48109, USA; b Department of Integrative Biology, University of California, Berkeley, CA 94720, USA

**Keywords:** Inhaled drug delivery, Microcrystal engineering, Antibiotic synergy, Colistin, Niclosamide, Composite microparticles, Spray drying, Antisolvent precipitation

## Abstract

Niclosamide and colistin sulfate exhibit strong in vitro synergistic activity against multidrug-resistant Gram-negative pathogens in cystic fibrosis; however, their co-formulation for inhaled delivery is constrained by differences in physicochemical properties and the high drug loading required for activity. Here, we report a formulation-focused strategy to enable excipient-minimized pulmonary co-delivery of niclosamide with colistin sulfate by engineering microparticles through liquid antisolvent precipitation followed by spray drying. Colistin sulfate was intentionally leveraged as a surface-active crystallization excipient, functioning both as an antimicrobial agent and as an interfacial stabilizer during niclosamide microcrystal formation. By varying the colistin: niclosamide molar ratio, median niclosamide particle size could be tuned from ~7.7 μm at 1:1 to ~1.3 μm at 24:1. Colistin adsorption inverted particle surface charge and suppressed time-dependent growth, consistent with interfacial stabilization during crystallization. The COL-NIC 8:1 formulation was downstream-processed via spray drying (geometric median size = 1.46 ± 0.50 μm) and exhibited favorable aerosol performance when delivered using a medium-resistance dry powder inhaler (FPF < 5 μm 75.3 ± 1.0%). Solid-state characterization indicated that niclosamide retained its crystalline structure, with no evidence of strong interaction with colistin. Finally, a preliminary in vivo lung infection model was used to assess the feasibility of pulmonary administration and the antibacterial response of the co-processed formulation relative to a standard-of-care comparator. Together, these results establish a particle-engineering platform for producing high-drug-loading niclosamide-colistin inhalation powders, highlighting how one active ingredient can function as a processing aid for excipient-minimized co-delivery of antibiotic combinations.

## 1. Introduction

Synergistic antibiotic combinations remain a practical strategy for treating multidrug-resistant infections, particularly when the development of new chemical entities is slow or clinically risky. These combinations are typically identified in vitro under controlled conditions in which both agents are maintained within the same microenvironment at defined concentrations. In vivo, however, achieving coordinated exposure at the site of infection is complicated by differences in physicochemical properties and tissue interactions that can influence drug distribution and presentation.

Chronic, recurrent infections caused by multidrug-resistant Gram-negative opportunistic pathogens pose a persistent challenge in cystic fibrosis disease management. Structural and physiological alterations in the cystic fibrosis lung create microenvironments complicate effective antibiotic delivery (Filkins and O’Toole, 2015). As resistance continues to rise, strategies that enable reliable co-deposition of combination therapies directly to the airways are of increasing interest.

Colistin is a last-resort antibiotic for treating pulmonary Gram-negative infections in cystic fibrosis, but increasing resistance is reducing treatment options (Bonyadi et al., 2022; Delgarm Shams-Abadi et al., 2023; Sasal et al., 2025). While niclosamide lacks stand-alone activity against these pathogens, its combination with colistin has been shown to exhibit strong in vitro synergy against multiple Gram-negative pathogens (Domalaon et al., 2019; Copp et al., 2020; Ayerbe-Algaba et al., 2018; Romero-Gonzalez et al., 2025). Direct co-delivery of these drugs by inhalation offers a means to localize combination therapy to the airways while minimizing reliance on systemic administration. The inhaled route is clinically established in cystic fibrosis, where patients are accustomed to aerosolized therapies, and inhaled formulations of each drug have precedent in human use (Backer et al., 2021; European Medicines Agency).

Several constraints exist in the co-formulation of colistin sulfate and niclosamide for inhalation. Colistin sulfate is a highly water-soluble, polycationic peptide with surface-active properties (PubChem, 2026a; Wallace et al., 2010), whereas niclosamide is a poorly water-soluble, weakly acidic small molecule (PubChem, 2026b), making conventional single-phase spray drying or solution-based co-formulation approaches technically challenging. Additionally, colistin sulfate exhibits minimum inhibitory concentrations (MICs) in the low-to-mid μg/mL range against many Gram-negative pathogens, even when combined with niclosamide. (Ayerbe-Algaba et al., 2018; Romero-Gonzalez et al., 2025; Domalaon et al., 2019) For inhaled antibiotics with such potency, achieving therapeutically relevant concentrations within the airways requires delivery of milligram-scale doses, reinforcing the importance of high drug loading and minimizing non-therapeutic excipient mass.

Although a niclosamide–colistin composite nanoemulsion has recently been reported (Zhang et al., 2024), the substantial excipient content required for nanoemulsion stabilization limits achievable drug loading and may increase inhalation burden. These considerations highlight the need for an alternative formulation strategy capable of co-processing both agents into high-drug-loading particles without reliance on conventional stabilizers.

Prior work from our group highlighted that the physicochemical presentation of niclosamide influences combination performance under simulated inhalation-relevant conditions (Romero-Gonzalez et al., 2025). These findings suggested that controlling the solid-state form and crystal size of niclosamide could provide a means to modulate its presentation in co-delivery systems. Accordingly, this study employs a microcrystalline form of niclosamide, drawing on principles established in long-acting injectable formulations, where crystal size and solubility serve as design variables governing drug release and presentation (Shah and Hong, 2022).

We hypothesized that colistin sulfate could modulate and stabilize niclosamide crystal formation during antisolvent precipitation, enabling the production of controlled-size microcrystals that could be processed into high-drug-loading respirable powders by spray drying. This strategy leverages one active pharmaceutical ingredient as a dual-function component during particle fabrication, serving both as a therapeutic agent and as a surface-active crystallization modulator. By exploiting the intrinsic polycationic and interfacial properties of colistin, this approach enables excipient-minimized co-processing of two drugs into a single respirable particle without reliance on conventional stabilizers or carriers.

In this study, we describe the development and physicochemical characterization of an excipient-minimized inhalable niclosamide–colistin formulation produced via antisolvent precipitation and spray drying. Particle size, solid-state properties, and aerosol performance were evaluated to assess the feasibility of generating high-drug-loading respirable powders suitable for pulmonary delivery. A murine lung infection model was used as a benchmark to evaluate pulmonary administration and resulting bacterial burden relative to vehicle and a systemically administered standard-of-care comparator, rather than to evaluate in vivo synergistic interactions. Together, these studies establish a formulation framework for co-delivering two antibiotics to the lung and provide a foundation for future optimization and mechanistic evaluation.

## 2. Materials and methods

### 2.1. Generation of niclosamide particles via liquid antisolvent precipitation

A liquid antisolvent precipitation (LAP) was developed in-house to produce suspensions of niclosamide microparticles suitable for downstream processing into niclosamide-colistin sulfate composite powders. In LAP, a drug solution is introduced into a miscible antisolvent in which the drug is poorly soluble, producing rapid supersaturation, nucleation, and subsequent crystal growth. Mixing conditions and the presence of a stabilizing excipient influence these processes and therefore the resulting particle-size distribution.

Anhydrous ethanol (Fisher Scientific) was used as a solvent for niclosamide (Cayman Chemicals). The niclosamide concentration was fixed at 11 mg/mL. The antisolvent consisted of either Milli-Q^®^ ultrapure water (Merck Millipore) adjusted to pH 5.8 or a 100 mM sodium phosphate buffer adjusted to pH 5.8, 7.0, 8.0. In all experiments, the antisolvent volume was fixed at 25 mL and contained in a 150 mL Pyrex beaker. When colistin sulfate was evaluated as a stabilizer, it was added to the antisolvent at a free-base equivalent concentration of 1.5 mg/mL, below the reported critical micelle concentration (1.7 mg/mL) (Wallace et al., 2010). Antisolvent temperature effects were assessed by conducting precipitation either at ambient temperature or with the beaker placed in an ice bath.

**Fig. 1.**
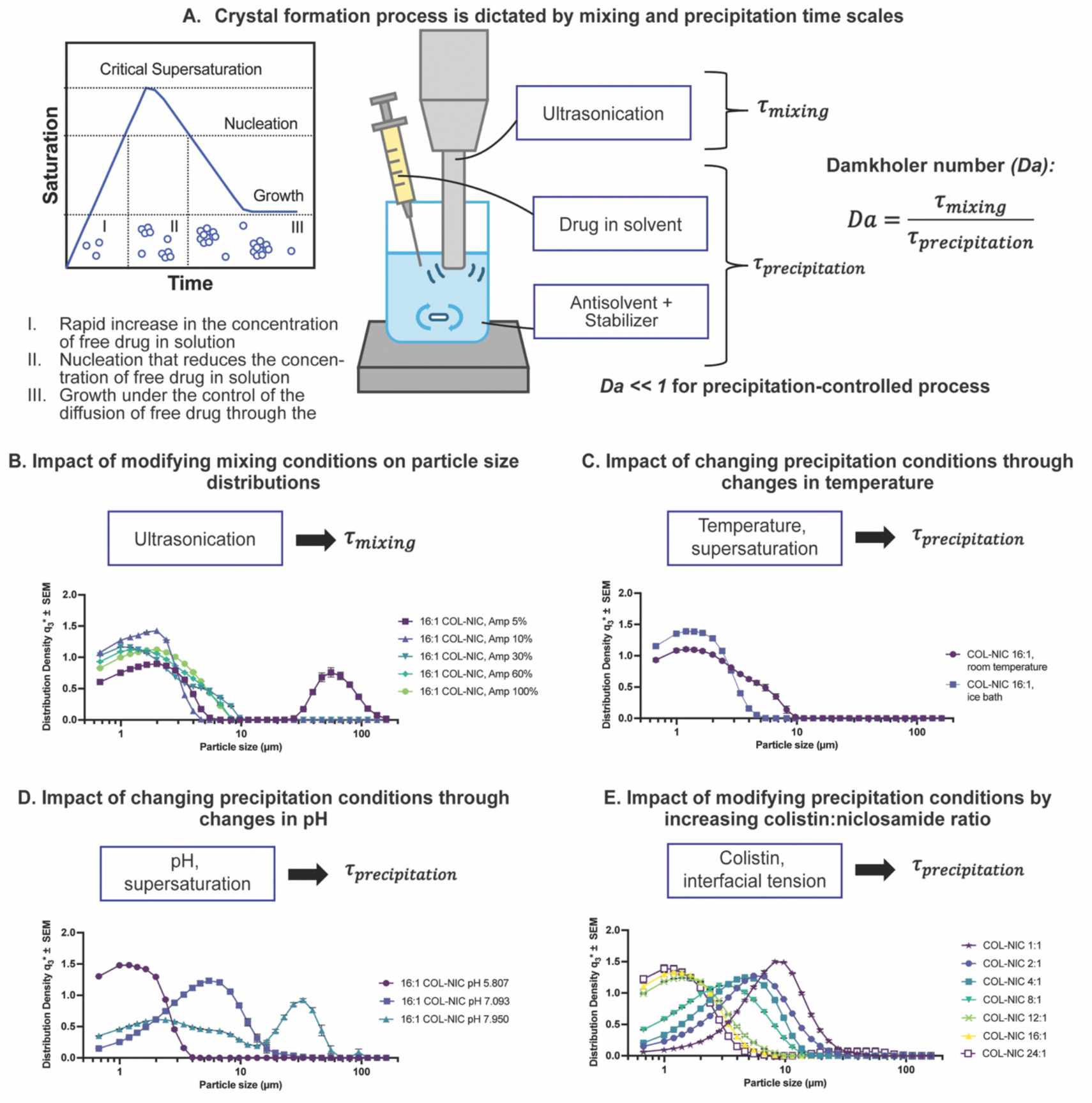
Process parameters evaluated for niclosamide microparticle formation. **(A)** Schematic overview of processing variables examined, including mixing conditions (ultrasonication amplitude) and precipitation conditions (temperature, antisolvent pH, and colistin:niclosamide molar ratio). **(B)** Effect of ultrasonication amplitude on particle size distributions. **(C)** Effect of precipitation temperature, comparing room-temperature and ice-bath conditions. **(D)** Effect of antisolvent pH on particle size distributions. 0.1 M sodium phosphate buffer at each reported pH was used as antisolvent **(E)** Effect of colistin:niclosamide molar ratio on particle size distributions, using 0.1 M sodium phosphate buffer pH 5.8 as antisolvent.

Method development focused on systematically varying mixing parameters, precipitation conditions (pH, temperature), and colistin concentration to identify conditions that reliably produce narrowly distributed, micron-sized niclosamide particles (Fig. 1). For downstream processing, the optimized LAP conditions were as follows:

Niclosamide-ethanol solution was added manually to the antisolvent (Milli-Q^®^ water, pH 5.8, with colistin, in an ice bath) using a 0.5 mL U-100 insulin syringe (29G × ½″), with injection volumes up to 1 mL based on the targeted niclosamide-to-colistin ratio. Mixing was provided by a 1-inch × 3/8-inch cylindrical stir bar placed at the bottom of the beaker and operated at 210 rpm. An ultrasonic probe (Fisherbrand CL-334) positioned 0.5 cm below the antisolvent–air interface supplied additional mixing. Probe amplitude was set to 10% of the instrument’s maximum output to assess the impact of sonication intensity on particle formation. Sonication and stirring were applied for 2 min in all experiments. Power and energy delivery were recorded for each batch.

### 2.2. Particle size and zeta potential characterization of niclosamide precipitates

Particle size distributions of the niclosamide suspensions resulting from the LAP process were measured by laser diffraction (Sympatec HELOS/R, CUVETTE wet dispersion unit, 50 mL quartz cuvette). Water (adjusted to pH 5.8) was used as the dispersion medium, and the niclosamide suspension was added to the cuvette until a measured optical concentration of at least 3% was achieved. Samples were maintained under stirring at 100 rpm during measurements. Each measurement was collected over a 10-second acquisition window and repeated in triplicate. Raw diffraction intensities were processed using Sympatec PAQXOS software, applying the FREE scattering model to obtain volume-weighted particle size distributions. Distribution data were plotted to determine $x_{10}$, $x_{50}$, and $x_{90}$, and the span was calculated as follows:

$$\text{Span}=\frac{x_{90}\;-\;x_{10}}{x_{50}}$$

Surface charge was quantified by zeta potential measurements using a Zetasizer Nano-ZS90 (Malvern Instruments). Suspensions were prepared identically to the particle-size experiments. Niclosamide-only control suspensions were generated by injecting the ethanolic niclosamide solution into pH 5.8 water in the absence of colistin. For each measurement, 1 mL of the suspension was transferred to an SCC-1.000 cuvette and equilibrated for 2 min before analysis using the Universal “Dip” Cell Kit (ZEN1002). Each sample was measured in triplicate, and the cuvette and dip cell were rinsed thoroughly with water and dried with a lint-free tissue between measurements to avoid cross-contamination.

### 2.3. Preparation of spray-dried niclosamide-colistin sulfate powder

Freshly prepared colistin-sulfate stabilized niclosamide microparticles (ten 25 mL batches pooled to a total volume of 250 mL) were spray-dried using a Buchi B-290 Mini Spray Dryer equipped with an B-290 inert loop and B-296 dehumidifier. The spray-drying feed consisted of the aqueous suspension generated by the antisolvent precipitation process; therefore, any residual organic solvent in the final powder would arise from ethanol carryover from the upstream precipitation step rather than from the primary spray-drying vehicle. The particle suspension was gently stirred throughout the process to prevent settling. Spray drying was carried out at an inlet temperature of 130 °C with 100% aspiration. The atomization nitrogen gas flow was set to 40 mm Hg on the rotameter, and the inert loop solvent condenser was maintained at 6 °C. The feed suspension was delivered via a peristaltic pump at 4 mL/min. The outlet temperature resulting from these conditions was ~70 °C. Spray-dried powder was collected and weighed to calculate the process yield, based upon the mass of solids in the feed.

### 2.4. *Niclosamide quantification by UV*–*Vis spectroscopy*

A niclosamide calibration curve was prepared to enable quantification by UV–Vis spectroscopy. A 0.5 mg/mL niclosamide stock solution was diluted in ethanol to prepare a series of dilutions, yielding standard concentrations ranging from 1.25 to 80 μg/mL. 200 μL of each standard solution (n = 3) was transferred into wells of a UV-transparent 96-well plate (Corning 3635), and absorbance was recorded at 330 nm. Blank-corrected absorbance values were averaged for each concentration, and the resulting data were fitted by linear regression to generate the niclosamide calibration curve.

### 2.5. Characterization of spray-dried niclosamide-colistin sulfate powder

#### 2.5.1. Niclosamide loading determination

Because the spray-dried formulation was intentionally designed as a binary system composed only of niclosamide and colistin sulfate, quantification of niclosamide following complete dissolution of the powder allowed direct determination of niclosamide loading, with the remaining mass corresponding to colistin sulfate by formulation design. Niclosamide loading in the spray-dried powders was therefore quantified by ethanol extraction followed by UV–Vis spectroscopy. Approximately 3 mg of powder was weighed into 1.5 mL microcentrifuge tubes (n = 5) and extracted with 1.5 mL of ethanol. A blank containing 3 mg of colistin sulfate in ethanol was prepared in parallel to represent the maximum expected colistin mass present during extraction and to confirm that colistin does not contribute measurable absorbance at 330 nm. Samples were sonicated for 5 min and centrifuged at 14,000 rpm for 10 min to sediment suspended colistin. Aliquots (200 μL) of each supernatant were transferred to a UV-transparent 96-well plate, and absorbance at 330 nm was recorded. Blank-corrected absorbance values were converted to niclosamide concentrations using the previously described calibration curve. Loading efficiency was calculated as:

$$Loading\;efficiency(\%)=\frac{\;\mathrm{Measured}\;\mathrm{loading}\;}{\mathrm{Theoretical}\;\mathrm{loading}}\times 100$$

#### 2.5.2. Particle size distribution of spray-dried powders (dried and reconstituted)

Dry-state particle size distributions were measured using a Sympatec HELOS laser diffractometer equipped with a RODOS disperser. Approximately 10–15 mg of spray-dried powder was introduced into the disperser at 3.0 bar. Three measurements were collected per sample. Diffraction data were analyzed using the same PAQXOS software parameters and FREE scattering model described in Section 2.2.

Wet-state particle size distributions were obtained by reconstituting spray-dried powders in PBS. Approximately 10 mg of powder was dispersed in PBS to yield a final solids concentration of 3.33 mg/mL, gently mixed by tube inversion, and transferred into the Sympatec CUVETTE containing ~40 mL of aqueous dispersion medium adjusted to pH 5.8. Suspensions were stirred at 1000 rpm and diluted until an optical concentration (Copt%) of ~3% was achieved. Reconstitution was performed using gentle mixing without high-shear dispersion, so the measured particle-size distribution reflects the extent to which niclosamide-containing clusters redisperse under low-energy conditions. Measurements were collected over a 10-second acquisition window and analyzed using the same software settings and calculation procedures described in Section 2.2.

#### 2.5.3. Scanning Electron Microscopy (SEM)

Powder morphology was examined using a JEOL JSM-7800FLV scanning electron microscope. Samples were gently dusted onto aluminum stubs coated with double-sided carbon tape and sputter-coated with ~10 nm of gold using a Denton Desk II sputter coater prior to imaging

#### 2.5.4. Solid state characterization of spray-dried powders

Solid-state properties of the spray-dried niclosamide–colistin sulfate powders were assessed by X-ray diffraction (XRD), modulated differential scanning calorimetry (mDSC), and attenuated total reflectance Fourier-transform infrared spectroscopy (ATR-FTIR). A physical mixture of unprocessed niclosamide and colistin sulfate, prepared by geometric dilution to match the mass ratio of the spray-dried formulation, was included as a reference control in all analyses.

##### X-ray diffraction (XRD).

Powders (spray-dried and physical mixture controls) were lightly ground, sieved through a 170-mesh screen, and analyzed on a Rigaku Ultima IV diffractometer using Cu Kα radiation. Samples were scanned in Bragg–Brentano geometry with a 2θ scan range of 5 to 90° at 2°/min to assess crystalline structure and detect changes resulting from spray drying.

##### Modulated Differential Scanning Calorimetry (mDSC).

Thermal transitions were evaluated on a TA Discovery 2500 DSC. Samples were loaded into Tzero pans with hermetically sealed lids, and a hole was pierced to prevent pan deformation. Samples were cooled to −40 °C at 10 °C/min, followed by a modulated heating ramp to 250 °C at 2 °C/min with a ± 0.5 °C modulation every 40 s. Data were processed using TA Universal Analysis.

##### Attenuated Total Reflectance Fourier Transform Infrared (FTIR-ATR) spectroscopy.

ATR-FTIR spectra were collected using a Nicolet iS50 FTIR spectrometer equipped with a diamond ATR accessory. Powder samples were pressed onto the crystal under constant pressure, and spectra were recorded from 4000 to 525 cm^−1^ at 4 cm^−1^ resolution (8 scans). Background spectra were collected prior to each measurement, and triplicate spectra were baseline-corrected and normalized using OMNIC software. For comparison of peak positions between samples, only regions with identifiable peaks in all replicates were analyzed. A threshold of ≥ 5 cm^−1^ was applied to distinguish spectral differences from typical instrumental variation and data-processing noise.

#### 2.5.5. Aerosol performance of spray-dried powders

Aerodynamic particle size distribution was assessed using a Next Generation Impactor (NGI; Copley Scientific) operated at 80 L/min, corresponding to a 4 kPa pressure drop across a medium-resistance RS01 inhaler (Plastiape S.p.a), with a total air volume of 4 L delivered over 3 s. Spray-dried niclosamide-colistin sulfate (8:1) powder was filled into HPMC Zephyr size 3 capsules (Lonza) at ~20 mg per capsule (n = 6 capsules; three NGI replicates). Capsule fill mass was determined gravimetrically.

Prior to testing, NGI collection cups were pre-coated with a 1% w/v glycerol solution in ethanol to minimize particle bounce and allowed to dry before use. Two capsule actuations were performed per NGI run. After actuation, residual formulation was recovered from the capsules, inhaler, induction port (IP), adapter, impactor stages, and micro-orifice collector (MOC) by rinsing with ethanol. Extracts were sonicated, centrifuged at 4000*g* for 5 min, and 200 μL aliquots of each supernatant were analyzed at 330 nm by UV–Vis spectroscopy using the niclosamide calibration curve described in Section 2.4. Mass balance was calculated as the total recovered niclosamide divided by the nominal loaded dose, with recoveries maintained within 80–120%.

Aerosol performance was evaluated using emitted fraction (EF) and fine particle fraction (FPF). EF was defined as the percentage of the total recovered niclosamide deposited downstream of the device. FPF < 5 μm and FPF < 3 μm were calculated as the percentage of the total recovered dose with aerodynamic diameters below 5 μm and 3 μm, respectively. NGI stage cut-off diameters were adjusted for the operating flow rate according to: $D_{50,Q}=D_{50,Q}{\left(\frac{Q_{n}}{Q}\right)}^{x}$ and the MOC cut-off diameter using:

$$D_{80,Q}=0.14{\left(\frac{Q_{n}}{Q}\right)}^{1.36}$$

where $D_{50,Q}$ is the cut-off diameter at the flow rate Q, $D_{50,Qn}$ is the cut-off diameter at the archival reference values of $Q_{n}=80\;L/min$, and the values for the exponent, x, are those obtained from the archival NGI stage cut size-flow rate calculations determined by Marple et al. (Marple et al., 2003)

### 2.6. Murine lung infection model for pulmonary delivery benchmarking

A murine lung infection model was used as a benchmark system to evaluate pulmonary delivery of the co-processed niclosamide–colistin formulation and its effect on lung bacterial burden relative to vehicle and a standard-of-care comparator. This benchmark model was intentionally designed to assess baseline delivery feasibility and antibacterial effects, not to evaluate synergistic interactions between the two agents. C3HeB/FeJ mice were intratracheally inoculated with *Burkholderia cenocepacia* isolate AU0728 using an established intratracheal infection procedure commonly employed for pulmonary infection studies in mice. 24 h post-infection, mice (n = 5 per group) received a single intratracheal dose of spray-dried COL-NIC (8:1) particles resuspended at 3.33 mg/mL in sterile phosphate-buffered saline (PBS), with 50 μL administered per mouse. This strain was selected based on ongoing parallel studies by our group demonstrating reproducible and sustained pulmonary infection with *B. cenocepacia* AU0728 relative to more resistant genetic backgrounds, enabling quantitative assessment of antibacterial effects. This corresponded to a total delivered powder dose of 8.3 mg/kg, containing approximately 0.24 mg/kg niclosamide. Control groups received 50 μL of sterile PBS intratracheally (negative control) or 180 mg/kg of ceftazidime administered intraperitoneally, prepared fresh in PBS (positive control). Doses were selected based on published reports (Pfefferle et al., 2023; Berkhout et al., 2015) and bacterial susceptibility to enable a pharmacologically relevant benchmarking comparison, noting that systemic (ceftazidime) and local (COL-NIC) routes produce different tissue exposures.

At 48 h post-infection, mice were euthanized, and lungs were aseptically harvested into 1 mL PBS and homogenized using bead disruption. Serial dilutions of lung homogenates were plated on nutrient agar, and bacterial burden was quantified as colony-forming units (CFU) per lung tissue. CFU counts were compared across treatment groups to assess relative antibacterial effects following pulmonary or systemic administration. Statistical analysis was performed on log10-transformed CFU values using one-way analysis of variance (ANOVA) with Dunnett’s multiple-comparison post hoc test versus vehicle-treated animals. A p-value < 0.05 was considered statistically significant.

## 3. Results

### 3.1. Effect of processing parameters on niclosamide particle size distributions

Niclosamide particle size distributions were evaluated across processing parameters spanning mixing-controlled and precipitation-controlled regimes (Fig. 1A). Ultrasonication amplitude, precipitation temperature, antisolvent pH, and colistin:niclosamide molar ratio were systematically varied, and the resulting particle size distributions are shown in Fig. 1B–D and Table 1.

Varying the ultrasonication amplitude significantly influenced the resulting particle sizes (Fig. 1B). Reducing the amplitude to 5% produced larger particles (x_50_ = 2.33 ± 0.13 μm), whereas 10% amplitude yielded smaller particles (x_50_ = 1.34 ± 0.00 μm). Amplitudes above 10% broadened the distribution (span_(10)_ = 1.48 ± 0.00; span_(30)_ = 2.87 ± 0.02).

The effect of temperature during precipitation was assessed by comparing room-temperature and ice-bath conditions (Fig. 1C). Ice-bath processing resulted in a smaller median particle size and narrower span (x_50_ = 1.27 ± 0.02 μm; span = 1.57 ± 0.04) than room-temperature processing (x50 = 1.57 ± 0.08 μm; span = 2.56 ± 0.17).

Particle formation was also evaluated across antisolvent pH values using phosphate buffer at pH 5.8, 7.0, and 8.0 (Fig. 1D). At pH 5.8, particles were the smallest and most narrowly distributed (x_50_ = 1.17 ± 0.01 μm; span = 1.43 ± 0.02). At pH 7.0, median size increased (x_50_ = 4.55 ± 0.24 μm; span = 1.98 ± 0.17), and at pH 8.0, distributions became broader and multimodal (x_50_ = 5.21 ± 0.11 μm; span = 6.94 ± 0.21).

Finally, the effect of colistin:niclosamide molar ratio on particle size was examined (Fig. 1E; Table 1). Median particle size decreased with increasing colistin content, with x_50_ values ranging from 7.66 ± 0.18 μm at a 1:1 ratio to 1.24 ± 0.09 μm at a 24:1 ratio (Table 1). Spans remained approximately 1.7–2.3 across most ratios, except at 24:1, where the span increased due to the presence of larger particles.

### 3.2. Effect of colistin sulfate on particle surface charge and growth stability

**Fig. 2.**
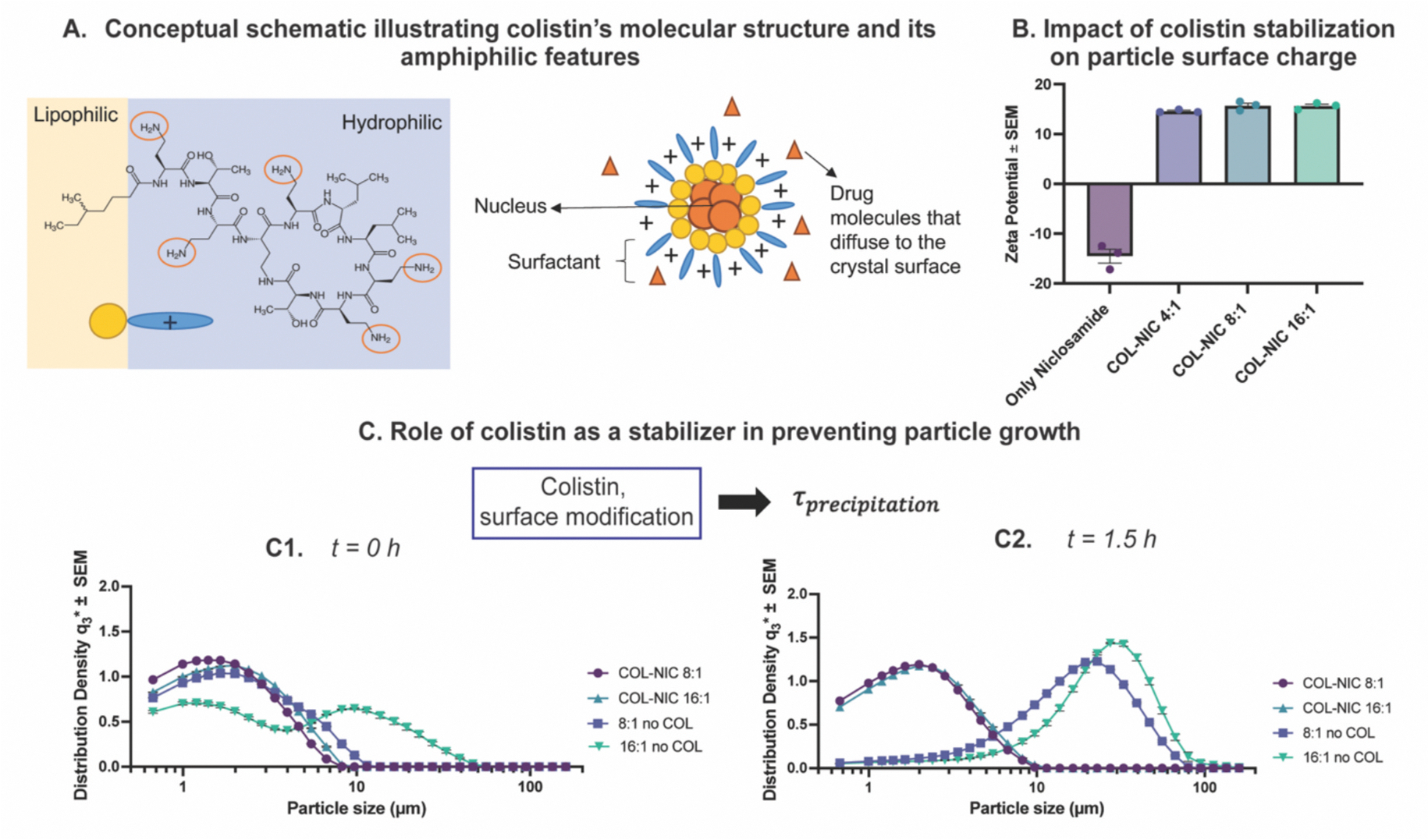
Characterization of particle surface charge and growth behavior in the presence or absence of colistin sulfate using water as antisolvent. **(A)** Conceptual schematic illustrating colistin’s molecular structure and spatial presentation around forming particles. **(B)** Zeta potential of niclosamide particles prepared with and without colistin. **(C)** Particle size distributions at t = 0h (**C1**) and t = 1.5 h (**C2**) for formulations prepared with colistin compared to colistin-free controls.

A schematic of colistin’s molecular structure, highlighting its amphiphilic regions, is shown in Fig. 2A. To assess how the presence of colistin relates to particle surface properties, the zeta potential of freshly prepared suspensions was measured (Fig. 2B). Particles prepared with colistin exhibited a positive zeta potential, whereas particles prepared in its absence were negatively charged.

Particle size distributions were monitored immediately after preparation (t = 0h) and after 1.5 h to evaluate changes in particle size over time (Fig. 2C; Table 2). At t = 0h, median particle sizes for colistin-containing formulations ranged from 1.49 to 10.03 μm depending on the molar ratio, while the corresponding colistin-free controls ranged from 1.49 to 17.28 μm. After 1.5 h, formulations prepared with colistin showed minimal changes in median particle size (fold increase of 1.08–1.77). In contrast, particles prepared without colistin showed larger increases in x_50_ (fold changes of 1.39–7.81), consistent with broader particle growth in the absence of stabilizer.

### 3.3. *Spray drying of niclosamide*–*colistin suspensions and characterization of the resulting powder*

**Fig. 3.**
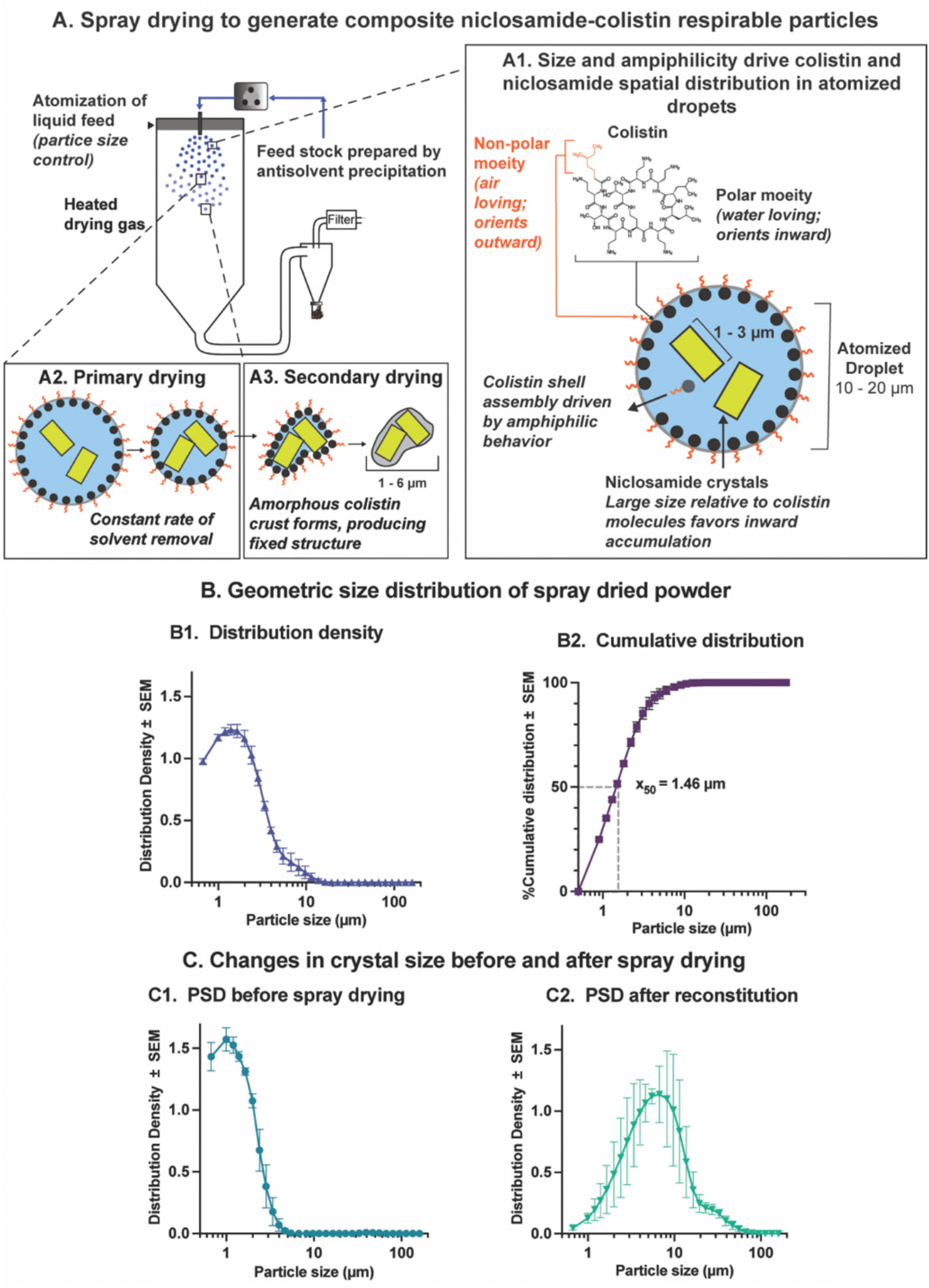
Spray drying of niclosamide-colistin suspensions and characterization of the resulting powder. (**A**) Conceptual schematic of the spray-drying process used to convert colistin-stabilized niclosamide suspensions into a dry powder. (**A1**) Atomization of the liquid feed into drying gas. (**A2–A3**) Solvent evaporation and formation of dried particles. (**B**) Geometric particle size distribution of the spray-dried powder, shown as distribution density (**B1**) and cumulative distribution (**B2**). (**C**) Particle size distributions of niclosamide suspensions before spray drying (**C1**) and after reconstitution of the spray-dried powder (**C2**). Data represent the average of three measurements from three independent batches; error bars indicate SEM.

Colistin-stabilized niclosamide suspensions generated by antisolvent precipitation were spray-dried to produce a respirable powder (Fig. 3A). The spray-drying yield was 42%, consistent with small-scale laboratory operation where proportional losses due to powder adhesion to drying chamber surfaces are common. Niclosamide loading in the dried powder was 2.83% ± 0.08% w/w, corresponding to a loading efficiency of 100.25% ± 2.8%.

The geometric particle size distribution of the spray-dried powder was unimodal (Fig. 3B1) with a median particle size (x_50_) of 1.46 ± 0.50 μm and a span of 2.23 ± 0.64 (Fig. 3B2). The size of the niclosamide crystals in suspension before spray drying and reconstituted after spray drying was measured using laser diffraction (Fig. 3C). Prior to spray drying, the suspended particles had an x_50_ of 1.11 ± 0.11 μm and a span of 1.45 ± 0.17 (Fig. 3C1). After spray drying and reconstitution, the median particle size increased to 6.01 ± 2.50 μm with a span of 2.21 ± 0.76 (Fig. 3C2). The larger particle size observed after reconstitution likely reflects incomplete redispersion of niclosamide microcrystals that were incorporated within the same spray-dried particle during droplet drying.

### 3.4. Solid state characterization of spray-dried niclosamide-colistin powder

**Fig. 4.**
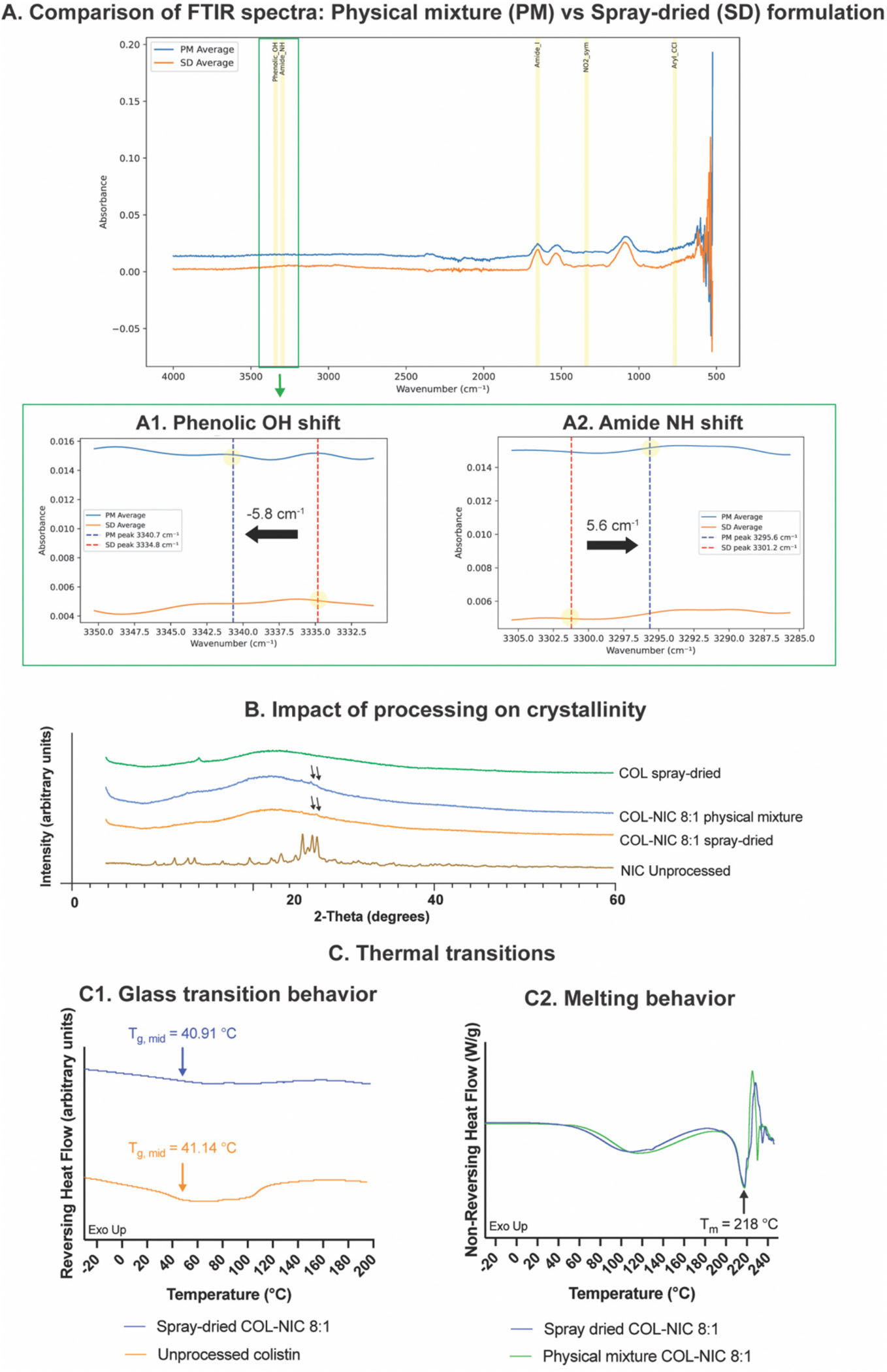
Solid-state characterization of niclosamide–colistin formulation. (**A**) FTIR spectra of the physical mixture (PM) and spray-dried (SD) niclosamide-colistin powder across the full wavenumber range. (**A1–A2**) Expanded views of selected vibrational regions showing measurable peak shifts in the phenolic O–H stretch (**A1**) and amide N–H stretch (**A2**). (**B**) X-ray diffraction patterns of unprocessed niclosamide (crystalline control), spray-dried colistin (amorphous control), the physical mixture, and spray-dried niclosamide-colistin powder. (**C**) Modulated differential scanning calorimetry thermograms. (**C1**) Glass transition behavior of spray-dried niclosamide-colistin powder compared to unprocessed colistin. (**C2**) Melting behavior of spray-dried niclosamide-colistin powder compared to niclosamide-colistin physical mixture.

FTIR spectra of the spray-dried niclosamide–colistin powder were compared with those of a physical mixture prepared at the same mass ratio (Fig. 4A). The spectra retained the characteristic vibrational bands of both components, indicating that no new covalent species were formed during processing. The phenolic O–H stretching vibration of niclosamide shifted from 3340.7 cm^−1^ in the physical mixture to 3334.8 cm^−1^ in the spray-dried formulation (Δ = −5.8 cm^−1^), while the amide N–H stretching band associated with colistin shifted from 3295.6 cm^−1^ to 3301.2 cm^−1^ (Δ = +5.6 cm^−1^) (Fig. 4A1–A2). These small shifts are consistent with changes in local hydrogen-bonding environment during co-processing rather than the formation of new chemical interactions.

Unprocessed niclosamide exhibited sharp diffraction peaks consistent with its crystalline structure, whereas spray-dried colistin alone produced a broad halo characteristic of an amorphous material. Both the physical mixture and the spray-dried niclosamide–colistin powder displayed weak reflections corresponding to niclosamide crystallinity. The reduced peak intensity in the spray-dried formulation is expected given that niclosamide represents only ~2.8% w/w of the composite powder, and the dominant amorphous colistin matrix contributes a strong background signal. These observations are consistent with retention of a crystalline niclosamide phase dispersed within an amorphous colistin matrix.

Modulated DSC analysis (Fig. 4C) further supported the coexistence of amorphous colistin and crystalline niclosamide phases. The reversing heat-flow signal revealed a glass transition at approximately 41 °C, consistent with the Tg of colistin (Fig. 4C1). In contrast, the non-reversing heat-flow signal exhibited a melting endotherm at 218 °C, corresponding to the melting of crystalline niclosamide (Fig. 4C2). The presence of this melting transition confirms that niclosamide remains crystalline after processing, consistent with the weak PXRD reflections observed in Fig. 4B.

### 3.5. Particle morphology and aerosol performance of spray-dried niclosamide-colistin powder

**Fig. 5.**
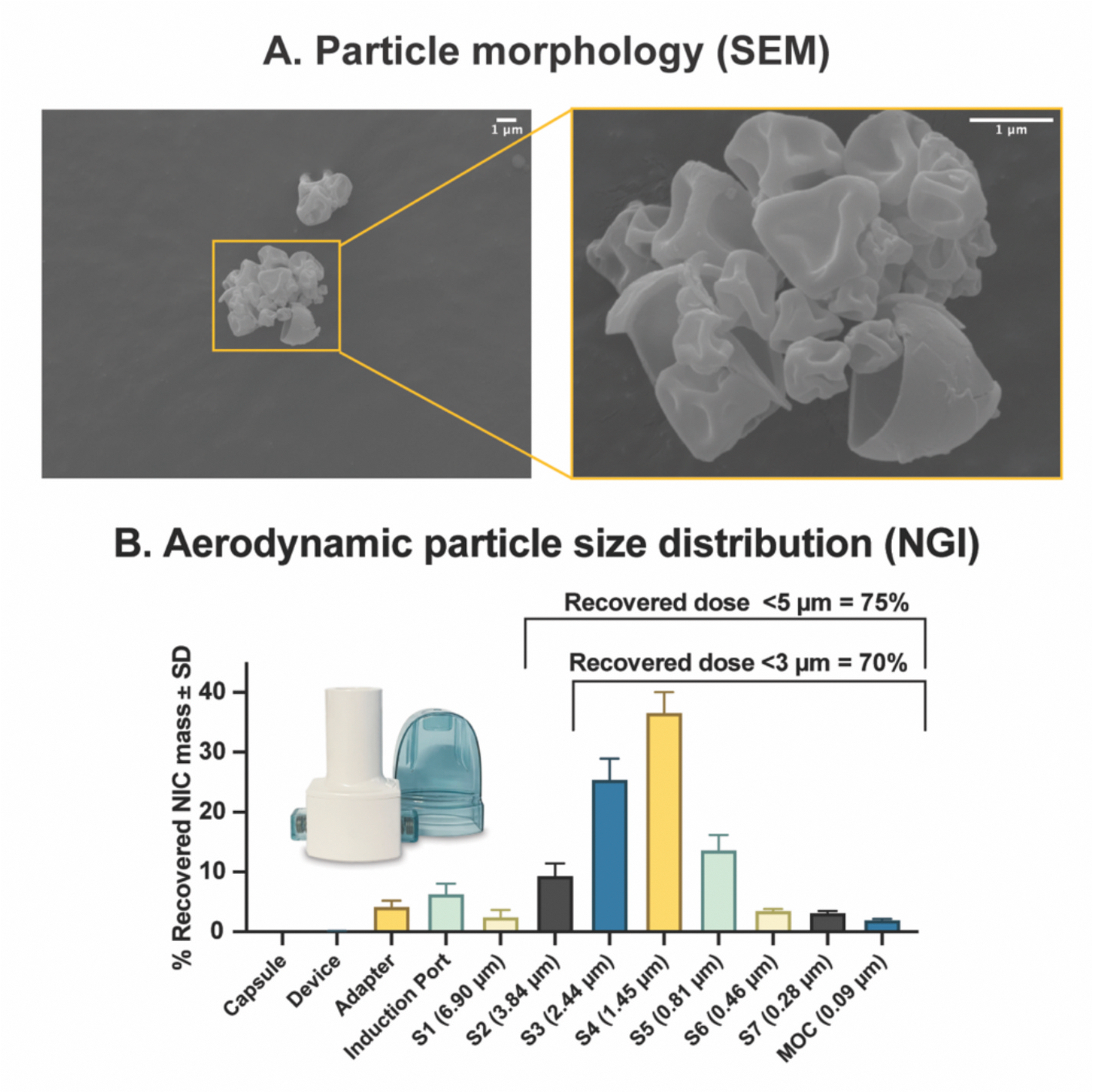
Morphology and aerodynamic performance of spray-dried niclosamide–colistin powder. (**A**) Scanning electron micrographs of the spray-dried powder showing collapsed, wrinkled particles. (**B**) Next Generation Impactor (NGI) deposition profile of the emitted dose, with recovered niclosamide mass across impactor stages corresponding to aerodynamic diameters < 5 μm and < 3 μm. Measurements were performed in triplicate (n = 3), and error bars represent the standard deviation.

SEM indicated that the spray drying process produced collapsed wrinkled particles (Fig. 5A). Aerodynamic particle size distribution was assessed using the Next Generation Impactor (Fig. 5B). The emitted fraction (EF%), defined as the percentage of the total recovered dose that was collected, was 85.9% ± 1.6%. The fine particle fraction (FPF < 5 μm) was 75.3% ± 1.0%. Aerodynamic deposition was quantified by measuring niclosamide recovered from each impactor stage following complete dissolution of the collected powder. Because the spray-dried formulation contains only two components, niclosamide and colistin sulfate, direct quantification of niclosamide provided confirmation of drug incorporation within the respirable composite particles. However, aerodynamic fractionation of each API was not independently measured, and therefore potential differences in the aerodynamic distribution of niclosamide and colistin sulfate across particle size fractions cannot be excluded. Independent quantification of both APIs across aerodynamic fractions will be required in later-stage development.

### 3.6. Pulmonary delivery of spray-dried colistin-niclosamide particles reduces lung bacterial burden in a murine infection model

**Fig. 6.**
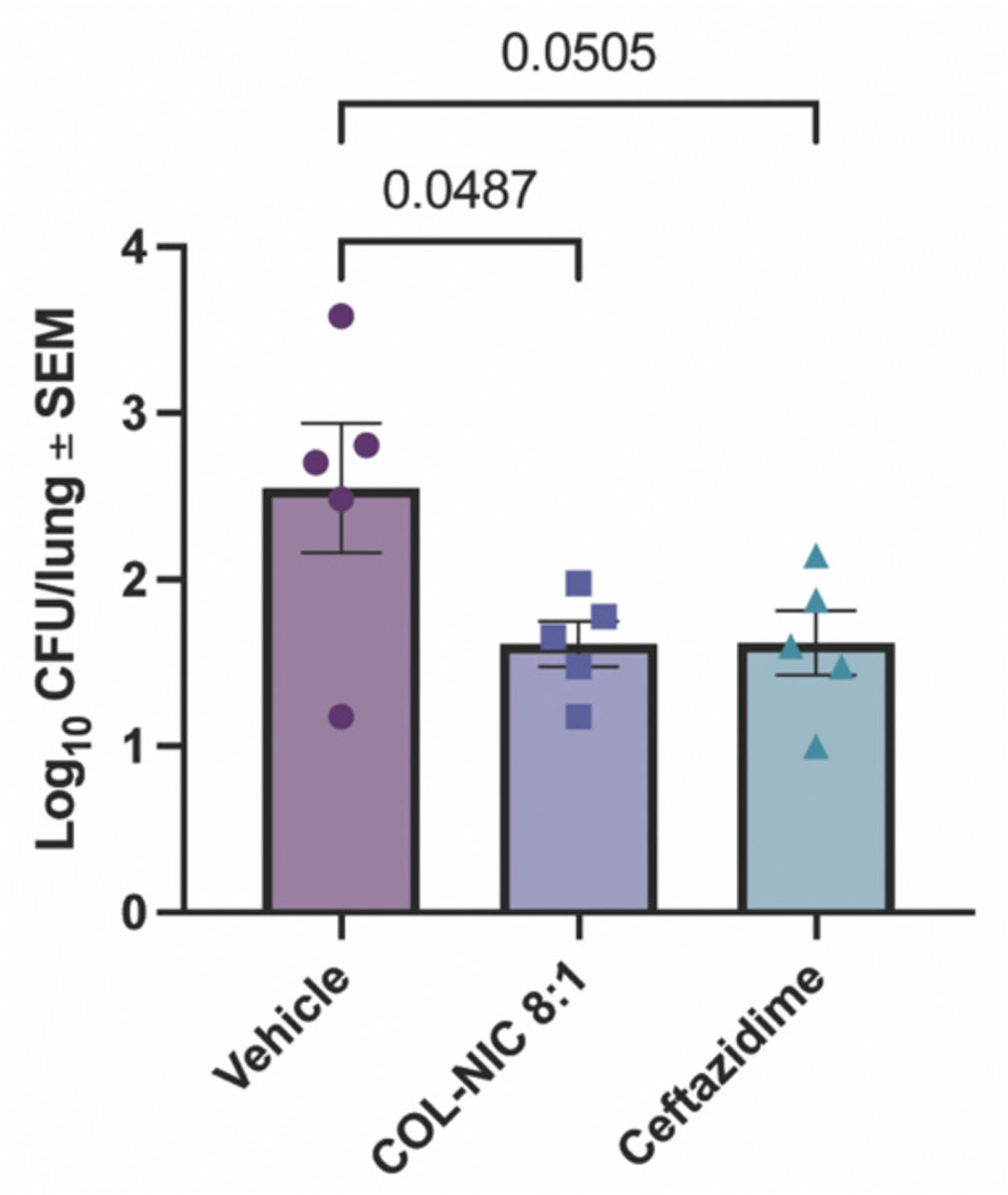
Lung bacterial burden following treatment with vehicle control (PBS), COL–NIC 8:1 spray-dried particles, or ceftazidime (standard-of-care comparator). Bacterial counts are reported as colony-forming units (CFU) per lung. Each data point represents an individual animal, with bars indicating mean ± SEM. Statistical analysis was performed using one-way ANOVA followed by Dunnett’s multiple-comparison test versus vehicle control.

In a murine lung infection model, intratracheal administration of the spray-dried colistin-niclosamide particle formulation resulted in a statistically significant reduction in lung bacterial burden relative to vehicle-treated animals (one-way ANOVA with Dunnett’s post hoc test, p = 0.0487). Treatment with ceftazidime produced a numerical reduction in CFU per lung compared to vehicle; however, this difference did not reach statistical significance after correction for multiple comparisons (p = 0.0505). Notably, the magnitude of bacterial burden reduction observed with the niclosamide-colistin formulation was comparable to that observed with the standard-of-care comparator in this benchmark study (Fig. 6).

## 4. Discussion

A primary goal of this work was to establish a controllable method for generating niclosamide microcrystals that could be incorporated into high-drug-loading composite particles suitable for respirable delivery. A central mechanistic insight that emerged from this study is that colistin actively participates in the crystallization process: its amphiphilic, cationic structure enables adsorption onto nascent niclosamide surfaces, lowering interfacial energy and limiting particle–particle fusion. This colistin-mediated surface stabilization supported robust crystal formation and contributed to the stability of the composite particles during spray drying and subsequent redispersion.

At the infinitesimal scale, crystal nucleation is governed solely by the local degree of supersaturation, which determines whether the free-energy barrier for nucleus formation can be overcome (Turnbull and Fisher, 1949). Antisolvent precipitation leverages this principle by rapidly reducing solubility through dilution into a poorer solvent, thereby generating the supersaturation needed to initiate nucleation.

For weakly acidic compounds such as niclosamide (pK_a_ ≈ 6.9), the ionization state strongly governs solubility and therefore the attainable supersaturation. In our antisolvent system, lowering the aqueous-phase pH increases the fraction of protonated niclosamide (Needham, 2022). Consequently, decreasing pH reduces the equilibrium solubility ($C^{*}$), raises the supersaturation ratio $\beta =\frac{C_{0}}{C^{2}}$, and strengthens the thermodynamic driving force for nucleation. Consistent with this framework, lower pH values produced smaller and more uniform particles under otherwise identical conditions, reflecting the more rapid and spatially extensive nucleation expected from higher supersaturation.

While the local supersaturation governs nucleation at the infinitesimal scale, supersaturation in real antisolvent precipitation is dictated by system-scale mixing (Thakur et al., 2022). Mixing kinetics govern the spatial and temporal profile of supersaturation (Rodríguez-Hornedo and Murphy, 1999). The interplay between supersaturation formation and nucleation can be expressed in terms of the characteristic timescales for mixing and precipitation (Matteucci et al., 2006). The mixing time, $\tau_{mix}$, describes how rapidly solvent and antisolvent interdiffuse to generate supersaturation, whereas the precipitation time, $\tau_{precip}$, reflects the kinetics of nucleus formation and early crystal growth. The relative magnitudes of these timescales determine the crystallization regime and are commonly captured by the Damköhler number: (Matteucci et al., 2006)

$$Da=\frac{\tau_{mix}}{\tau_{precip}}.$$

When Da<1, supersaturation is established more rapidly than precipitation, producing a homogeneous burst of nuclei and narrow particle size distributions. By contrast, when Da>1, mixing is slower than precipitation, supersaturation develops unevenly, and nucleation becomes spatially localized, yielding broader or multimodal size distributions (Matteucci et al., 2006). This timescale framework provides a quantitative basis for interpreting how process conditions shape the breadth and uniformity of the resulting niclosamide crystals.

Hydrodynamics in our system were governed by three main inputs: injection rate, stirring speed, and ultrasonication energy. Only ultrasonication intensity was varied. In liquid systems, mixing arises from the formation and decay of eddies that transport solvent and antisolvent across multiple length scales (Thakur et al., 2022). The resulting mixing time can be decomposed into mesomixing and micromixing contributions,

$$\tau_{mix}=\tau_{meso}+\tau_{micro}.$$

Because injection rate and stirring were held constant, $\tau_{meso}$ was not expected to vary appreciably as it reflects the convective exchange and breakup of larger eddies generated during antisolvent injection and bulk agitation.

Micromixing corresponds to the final homogenization of concentration gradients at molecular scales and depends strongly on the local energy dissipation rate, ε:

$$\tau_{\text{micro}}=k_{m}{\left(\frac{\nu }{\varepsilon }\right)}^{1/2}.$$

Higher ε accelerates the breakdown of small eddies and shortens $\tau_{micro}$. Ultrasonication disproportionately affects this regime because acoustic cavitation injects energy directly at small hydrodynamic length scales; collapsing cavities generate shock waves, microjets, and regions of very high shear that rapidly eliminate composition gradients and increase ε (Dalvi and Dave, 2009). Cavitation-induced dissipation therefore reduces $\tau_{micro}$, favoring rapid and spatially uniform supersaturation development.

While increasing sonication amplitude reduces $\tau_{micro}$, it also generates heat, raising the local solution temperature. Because niclosamide aqueous solubility increases with temperature (Needham, 2022), this thermal erosion of supersaturation counteracts the benefits of faster micromixing (Rodríguez-Hornedo and Murphy, 1999). As a result, ultrasonication exhibits an upper practical limit: intermediate amplitudes produced the narrowest particle size distributions, whereas higher amplitudes broadened them. Cooling the antisolvent, which lowers solubility and increases supersaturation, restored narrow distributions under otherwise identical conditions. Once supersaturation has been established through these mixing- and energy-dissipation processes, the subsequent stability and evolution of newly formed nuclei are dictated by interfacial thermodynamics.

Creating new surface area raises the free energy of a system because molecules at an interface experience incomplete coordination and therefore contribute an excess surface free energy γ (Gibbs, 1878). As nuclei become smaller, this surface contribution dominates their total free energy, making them increasingly unstable. The equilibrium chemical potential of a condensed phase increases with curvature, such that phases bounded by highly curved (small-radius) interfaces require higher equilibrium vapor pressure, and by analogy, higher solubility, than those with planar interface (Thomson, 1872). This curvature dependence, formalized in the Gibbs–Thomson relation, predicts that small particles possess elevated chemical potential and therefore tend to dissolve or fuse to reduce interfacial area. This thermodynamic penalty drives Ostwald ripening: particles below a critical radius dissolve while larger particles grow at their expense, coarsening the population to minimize total γA (Lifshitz and Slyozov, 1961). Because these thermodynamic forces act immediately after nuclei are formed, the earliest particles evolve rapidly unless their surfaces are quickly passivated. These same curvature- and surface-energy–driven processes define the early-stage transformations captured within the precipitation time, $\tau_{\text{precip}}$, a period that encompasses not only nucleation but also condensation, growth, coalescence, and ripening (Matteucci et al., 2006).

Stabilizers intervene directly in interfacial thermodynamics, rendering newly formed nuclei unstable by lowering the interfacial free energy γ of the drug–water boundary and replacing high-energy drug–solvent contacts with more favorable drug–surfactant–solvent interactions. Because γ appears in both the surface free energy (γA) and in the curvature-dependent chemical potential described by the Gibbs–Thomson relation, its reduction diminishes the thermodynamic driving forces for fusion, dissolution, and Ostwald ripening. Adsorbed layers also introduce steric or electrostatic barriers that slow collision-induced coalescence and impede growth along high-energy crystal faces, effectively increasing the precipitation timescale, $\tau_{\text{precip}}$, shifting the system toward a lower effective Damköhler number and enabling narrow particle size distributions even under mixing conditions that would otherwise favor growth and aggregation (Matteucci et al., 2006; Dalvi and Dave, 2009). Colistin, traditionally viewed as only an antibiotic, possesses the molecular architecture of an effective crystallization excipient: its hydrophobic fatty acyl tail can anchor to hydrophobic drug surfaces, while its cyclic peptide ring presents multiple protonated amines that remain solvated and confer strong cationic charge. This amphiphilic arrangement is analogous to classical surfactants, enabling colistin to lower the interfacial free energy γ, reduce the curvature-dependent chemical potential of small nuclei, and introduce electrostatic barriers that inhibit particle–particle fusion. These predicted behaviors were observed experimentally. Increasing the colistin: niclosamide molar ratio progressively decreased particle size, consistent with enhanced nucleation and a reduced critical nucleus radius-effects commonly associated with surfactant-mediated γ reduction (Dalvi and Dave, 2009). Zeta potential measurements confirmed that colistin adsorbs to niclosamide crystal surfaces and inverts the surface from negative to positive, indicating that even low colistin concentrations are sufficient to establish a colistin-dominated interface. Beyond this threshold, additional increases in the colistin:niclosamide ratio did not substantially change the measured surface potential, consistent with near-saturation of the interfacial layer. In contrast, particle size continued to decrease over this same concentration range, reflecting the sensitivity of early-stage nucleation, coalescence, and ripening dynamics to the bulk stabilizer concentration rather than to the final plateaued zeta potential. These combined thermodynamic and kinetic effects increase the effective precipitation timescale, $\tau_{\text{precip}}$, shifting the system toward a lower Damköhler number and enabling narrow size distributions even under fixed mixing conditions (Matteucci et al., 2006). Consistent with this interpretation, particles prepared without colistin underwent rapid ripening and broadening over hours, whereas colistin-stabilized particles remained stable. Together, these results demonstrate that colistin functions as a surface-active excipient capable of shaping the early crystallization environment and governing the evolution of particle size. The colistin:niclosamide ratios used here were within clinically deliverable inhaled dose ranges for each drug individually, based on approved products and human studies (Backer et al., 2021; European Medicines Agency; TFF-Pharmaceuticals, 2022).

FTIR analysis of the spray-dried downstream-processed formulation did not reveal the appearance of new bands or large vibrational shifts relative to the physical mixture, indicating that spray drying of the suspension prepared by antisolvent precipitation did not result in the formation of new chemical species or strong specific interactions between niclosamide and colistin. Both components, therefore, remain physically associated rather than chemically complexed. Modest shifts were nevertheless observed, including a small downshift (~6 cm^−1^) in the phenolic O–H stretch of niclosamide and a slight upshift (~6 cm^−1^) in the amide N–H stretch of colistin. These subtle changes likely reflect minor perturbations in the local hydrogen-bonding environment or molecular packing upon co-processing, confirming physical association through weak, non-specific intermolecular interactions.

When considered alongside XRD and thermal analysis, these FTIR results suggest a composite architecture in which crystalline niclosamide is dispersed within a predominantly amorphous, colistin-rich matrix, rather than forming a new solid phase. Because colistin sulfate constitutes the majority of the particle mass while niclosamide is present at low loading (~2.8% w/w), the overall diffraction and thermal signatures are strongly influenced by compositional dilution, which attenuates the intensity of niclosamide-specific features without indicating loss of crystallinity. The observed melting endotherm at 218 °C is consistent with reported melting temperatures for crystalline niclosamide (Jara et al., 2021), further supporting the retention of its crystalline structure following antisolvent precipitation and spray drying. In contrast, the presence of thermal degradation above 230 °C is consistent with the known thermal instability of colistin sulfate (Thermo Fisher Scientific, 2025). Collectively, these solid-state findings define the internal architecture of the spray-dried particles and provide a basis for understanding the particle-formation dynamics during drying.

This structural arrangement is consistent with particle-formation pathways predicted by the Péclet number framework for multicomponent spray drying (Vehring, 2008). Because colistin is a high-molecular-weight amphiphilic molecule with low diffusivity, it exhibits a high Péclet number and therefore migrates toward the droplet surface during solvent evaporation, forming a colistin-rich outer layer. In contrast, the niclosamide microcrystals suspended in the feed possess negligible diffusivity and remain confined within the droplet interior as drying progresses. The geometric size distribution of the spray-dried powder reflects these dynamics: final particle sizes do not directly map onto the initial niclosamide crystal distribution because individual droplets can contain a variable number of crystals, and droplet contraction during solvent removal brings multiple crystals into close contact, forming small multi-crystal agglomerates. Measurements across three independent spray-dried batches showed that dry powders were highly consistent, while resuspended powders displayed greater variability, reflecting differences in how multi-crystal clusters persisted upon rehydration. After resuspension in aqueous media, the amorphous colistin dissolves rapidly due to its high water solubility, leaving behind the poorly water soluble niclosamide crystals. The shift toward larger particle sizes observed upon reconstitution most likely reflects persistence of small multi-crystal clusters formed during droplet drying that were not fully dispersed under the low-energy reconstitution conditions used here. In the pulmonary environment, however, interactions with lung lining fluid and surfactant would be expected to promote dispersion of these clusters. Together, these data indicate that spray drying produces stable composite particles in which colistin forms the continuous phase and niclosamide is retained as discrete, occasionally clustered, microcrystalline domains.

A key limitation of this work is that antisolvent precipitation was conducted in an open, laboratory-scale system in which mixing and energy dissipation could only be adjusted coarsely through stirring speed, injection rate, and sonication amplitude. Because this configuration lacks the hydrodynamic control of engineered crystallization platforms, micromixing efficiency and spatial uniformity of supersaturation could only be optimized within a limited range. Consequently, the particle sizes and distributions reported here likely reflect the constraints of the laboratory setup rather than the theoretical limits imposed by nucleation and growth kinetics. While this level of control was sufficient to elucidate the mechanistic role of colistin in stabilizing nascent niclosamide nuclei, downstream functional performance will ultimately depend on how these early-stage processes influence niclosamide dissolution, release kinetics, and transport through airway mucus. Future work will therefore evaluate how formulation and process parameters, using systematic design-of-experiments approaches to interrogate critical material attributes and process variables, influence particle size distribution, release behavior, and mucus transport. Additionally, because aerodynamic deposition in this study was quantified using niclosamide as a tracer component, later-stage development will require independent aerodynamic fractional analysis of both active ingredients to confirm uniform distribution of niclosamide and colistin sulfate across aerodynamic size fractions.

To provide preliminary functional validation of the formulation, we benchmarked the spray-dried COL-NIC particles in a murine lung infection model. Intratracheal administration of the particles produced a statistically significant reduction in lung bacterial burden relative to vehicle-treated animals, with an effect magnitude comparable to that observed for systemic ceftazidime, despite substantial differences in dose and route of administration. These results indicate that the formulation can deliver therapeutically active concentrations of niclosamide and colistin to the lung, supporting the potential of excipient-minimized, high-drug-loading composite particles for local antibacterial activity. While this benchmark study was limited to a single-dose evaluation and did not include detailed pharmacokinetic measurements, and therefore cannot be used to evaluate synergistic interactions between the two agents, it provides proof-of-concept that co-processing niclosamide with colistin can yield an inhalable particle capable of reducing bacterial burden in vivo. Future studies will be required to quantify pulmonary exposure, optimize dosing, and assess long-term efficacy relative to conventional therapy.

## 5. Conclusions

This work establishes a controllable antisolvent precipitation strategy for generating niclosamide microcrystals and demonstrates that colistin sulfate can function as an effective crystallization excipient, stabilizing early nuclei and suppressing coalescence during particle formation. By tuning pH, temperature, and micromixing via ultrasonication, we identified conditions that reliably produce sub-2 μm niclosamide crystals suitable for downstream spray drying into high-drug-loading composite microparticles.

Spray drying these stabilized suspensions yielded respirable microparticles with a core–shell–like architecture in which crystalline niclosamide is embedded within an amorphous colistin-rich matrix. Structural analyses confirmed that niclosamide retains its crystallinity through drying and reconstitution, whereas colistin vitrifies and becomes surface-enriched, consistent with its predicted high Péclet number and amphiphilic character. Upon rehydration, colistin dissolves rapidly, liberating discrete niclosamide microcrystals and demonstrating physical decoupling of the two components without loss of structural integrity.

In a murine lung infection model, intratracheal administration of the niclosamide-colistin formulation reduced pulmonary bacterial burden relative to vehicle controls and achieved an effect size comparable to systemic ceftazidime, providing an initial in vivo benchmark of antibacterial activity for the engineered particles. These results support the feasibility of using this formulation strategy to deliver structurally defined niclosamide-colistin composites to the lung.

Together, these findings demonstrate that colistin can serve as both an antimicrobial agent and a surface-active excipient that governs crystal formation, particle morphology, and composite structure. The formulation principles established here provide a foundation for future studies that directly interrogate dissolution behavior, mucus interactions, and exposure–response relationships in more physiologically complex airway models.

## Figures and Tables

**Table 1:** Impact of increasing colistin:niclosamide molar ratio on median particle size and distribution span.

Colistin-to-niclosamide molar ratio	Median particle size x_50_ (μm) ± SD	Span ± SD

1:1	7.66 ± 0.18	1.72 ± 0.06
2:1	6.32 ± 0.01	1.86 ± 0.02
4:1	3.02 ± 0.01	2.02 ± 0.05
8:1	2.69 ± 0.05	2.21 ± 0.04
12:1	2.26 ± 0.05	2.35 ± 0.15
16:1	1.32 ± 0.07	1.81 ± 0.08
24:1	1.24 ± 0.09	6.71 ± 8.83

**Table 2:** Niclosamide particle growth in the presence or absence of colistin.

11 mg/mL NIC solution in ethanol injected (mL)	NIC mass introduced into antisolvent (mg)	Antisolvent composition	t = 0h Median particle size x_50_ (μm) ± SD	t = 0h Span ± SD	t = 1.5 h Median particle size x_50_ (μm) ± SD	t = 1.5 h Span ± SD	Fold increase in x_50_ from t = 0h to t = 1.5 h

1.00	11.0	1.5 mg/mL COL in water	10.03 ± 0.84	3.01 ± 1.43	11.30 ± 0.47	3.81 ± 0.17	1.12
		Water	17.28 ± 0.57	1.93 ± 0.01	26.35 ± 0.94	1.92 ± 0.00	1.52
0.25	2.75	1.5 mg/mL COL in water	1.82 ± 0.01	2.04 ± 0.01	3.22 ± 0.02	1.53 ± 0.02	1.77
		Water	1.49 ± 0.01	2.11 ± 0.02	11.64 ± 0.15	1.47 ± 0.01	7.81
0.12	1.32	1.5 mg/mL COL in water	1.49 ± 0.00	2.03 ± 0.01	1.73 ± 0.01	2.04 ± 0.01	1.16
		Water	1.83 ± 0.01	2.58 ± 0.02	17.11 ± 0.14	2.09 ± 0.02	9.35
0.06	0.66	1.5 mg/mL COL in water	1.70 ± 0.01	2.11 ± 0.01	1.85 ± 0.00	2.10 ± 0.01	1.08
		Water	4.06 ± 0.05	4.50 ± 0.18	14.15 ± 0.08	2.11 ± 0.01	3.49

## Data Availability

Data will be made available on request.
